# Current Methods for Recombination Detection in Bacteria

**DOI:** 10.3390/ijms23116257

**Published:** 2022-06-02

**Authors:** Anton E. Shikov, Yury V. Malovichko, Anton A. Nizhnikov, Kirill S. Antonets

**Affiliations:** 1 Laboratory for Proteomics of Supra-Organismal Systems, All-Russia Research Institute for Agricultural Microbiology (ARRIAM), 196608 St. Petersburg, Russia; a.shikov@arriam.ru (A.E.S.); yu.malovichko@arriam.ru (Y.V.M.); a.nizhnikov@arriam.ru (A.A.N.); 2 Faculty of Biology, St. Petersburg State University (SPbSU), 199034 St. Petersburg, Russia

**Keywords:** homologous recombination (HR), horizontal gene transfer (HGT), recombination detection, HGT detection, phylogenetic methods, synteny

## Abstract

The role of genetic exchanges, i.e., homologous recombination (HR) and horizontal gene transfer (HGT), in bacteria cannot be overestimated for it is a pivotal mechanism leading to their evolution and adaptation, thus, tracking the signs of recombination and HGT events is importance both for fundamental and applied science. To date, dozens of bioinformatics tools for revealing recombination signals are available, however, their pros and cons as well as the spectra of solvable tasks have not yet been systematically reviewed. Moreover, there are two major groups of software. One aims to infer evidence of HR, while the other only deals with horizontal gene transfer (HGT). However, despite seemingly different goals, all the methods use similar algorithmic approaches, and the processes are interconnected in terms of genomic evolution influencing each other. In this review, we propose a classification of novel instruments for both HR and HGT detection based on the genomic consequences of recombination. In this context, we summarize available methodologies paying particular attention to the type of traceable events for which a certain program has been designed.

## 1. Introduction

The bacterial genome is shaped by homologous recombination (HR) and horizontal or lateral gene transfer (HGT/LGT), with the latter represented by variable molecular mechanisms [1,2]. Recombination could be defined as an exchange of nucleotide sequences between different genomes or within a single genome [1]. If the donor sequence replaces the respective homologous (or homeologous, i.e., similar but not identical) region in the acceptor DNA molecule, then the process is called homologous recombination (HR) [3]. Broadly speaking, HGT could be defined as the incorporation of non-homologous genetic material into the donor genome which requires a long (>500 nucleotides) homologous region flanking the non-homologous segment [2,4]. During the incorporation, a direct RecA-dependent homologous recombination mediates the process, and it includes the excision of the transferred DNA fragment from the donor genome, and its integration into the recipient genome, implying two acts of homologous recombination. HR mostly affects core genes maintaining allelic diversity [5,6], while HGT induces the acquisition of accessory genes [7]. In bioinformatics literature, the term ”non-homologous recombination” (NHR) is sometimes used interchangeably with HGT [4,8], or NHR is seen as HGT-inducing machinery [9,10]; however, that is not always, if ever, true. In fact, DNA integration of mobile genetic elements into the recipient genome such as the integration of phages and genetic islands or conjugative transposons either by site-specific recombinases or by single-strand annealing proteins (SSAPs) requires micro-homologous and homologous sequences, respectively [11,12], that is, strictly speaking, this process could be treated as a type of homologous recombination. Nevertheless, it should be kept in mind that homologous recombination implies DNA strand exchange, whereas the integration processes mentioned do not include strand exchange. Therefore, in the current review by HR, we assume exchange between bacterial genomes and by HGT, we mean the incorporation of genetic material into the recipient genome driven by single-strand annealing (SSA) and/or site-specific recombination but not NHR. HR and HGT are interconnected with respect to the evolutionary dynamics of the bacterial genome. Horizontally transferred genes are often flanked by regions with a high HR rate [13] which could possibly maintain genome size by replacing/eliminating recently acquired genes [13,14]. Gene acquisition, loss, and replacement that are driven by HGT and HR often lead to the emergence of new pathogenic strains [15] and serotypes [16], including opportunistic pathogens [17], increased virulence [18], antibiotic resistance [19,20], immunity evasion [21,22], colonization of new hosts [23], and metabolic adaptations [24,25], thus, affecting public health.

Apart from practical implications, recombination exerts an effect on phylogenetic studies altering almost all trees’ parameters. Models applied in conventional phylogenetic analysis are based on the assumption that any parts of DNA or amino acid sequences determine the evolutionary history in the same way [26]. Nonetheless, if the data contain recombination events, the topologies of trees would differ depending on the part of the sequence, especially if the breakpoint is located in the middle of the sequence [1] which sometimes makes single locus-based phylogeny non-informative [27]. Furthermore, recombination exchange can result in terminal branches that are too long [28], loss of the molecular clock [28], non-uniform distribution of insertions and deletions [29], impossible to identify the common ancestor [30], and an erroneously high dN/dS ratio (the ratio of nonsynonymous to synonymous mutations) resulting in spurious signals of positive selection [31]. Using several housekeeping genes (5–20), namely, MLST (multilocus sequence typing) technique was proposed to overcome these issues; however, it cannot depict gene acquisition or replacement [5]. Progress in next-generation sequencing with high throughput has made it possible to use core genes in the genomes to reconstruct phylogenies, which is known as core genome MLST, or cgMLST. Unfortunately, it still cannot circumvent recombination-driven long terminal branches [32] or inaccurate topologies particularly when the selective pressure is high [33]. A prospective method to obtain trees with correct topology and branch lengths called the coarse-graining approach for phylogenetic reconstruction (CGP) has been devised recently, and it requires further studies to assess its effectiveness [34].

As stated above, HGT and HR are different, yet genomically connected processes. From a genomic perspective, it is virtually impossible to determine specific mechanisms and causes of a particular transfer and/or exchange event; therefore, researchers use indirect computational methods, namely, comparative genomics and phylogeny reconstruction. Here, we analyze state-of-the-art bioinformatics tools for detecting HGT and HR. We discuss conventional approaches as well as novel tools in the context of their pros and cons. We propose an integrated classification of the algorithms based on the ramifications of genetic exchanges, both HGT and HR. Finally, we examine major trends in modern tools’ designing new software and discuss the perspective of further developments.

## 2. A Brief Overview of Conventional Methods for Detecting Homologous Recombination and Horizontal Gene Transfer

Bioinformatics approaches for detecting genetic exchanges can be divided into several groups depending on the nature of the tasks set, applied algorithms, and genomic consequences that are analyzed. In the existing literature, researchers have separately discussed how to trace homologous recombination and HGT proposing distinct classifications. It is explainable as these two groups seem to have different goals: the former methods are aimed to calculate HR rates and detect chimeric loci in the closely related genomes [3,26], whereas the latter approaches reveal continuous genome regions, for example, genes or larger fragments, acquired from either related or evolutionary distinct species [2].

Considering the end goals of the analysis, methods for HR and HGT detection are divided depending on whether they accomplish: (i) revealing the evidence of exchanges/acquisitions, (ii) identifying mosaic sequences, (iii) finding breakpoint sites, or (iv) calculating recombination and HGT rates [3]. The first task is usually embedded into the latter ones; however, there are some algorithms designed only for revealing the fact of recombination in analyzed sequences applied mostly in HR studies. The second and the third goals are achieved by finding distinct local similarities among a subset of aligned sequences or via the identification of certain loci responsible for phylogenetic incongruences due to the exposure to recombination or horizontal transfer [26]. The last issue is mainly addressed by population genetics principles and phylogenetic analysis [35,36].

When describing the types of methods for HR analysis according to the statistical basis, it should be noted that they belong to so-called parametric and non-parametric methods. The former methods aims to calculate population parameters from a sample [3]. It implies revealing the average recombination frequency, which is achieved by population genetics methods based on a coalescent theory; therefore, these approaches assume the absence of selection and within-group subpopulations and constant population size [3]. The other methods rely on non-parametric statistics inferred directly from sequence alignments and/or tree topology [3]. A distinct methodology is reconstructing ancestral recombination graphs (ARGs) that include elements from all the aforementioned approaches and depict individual recombination events backed by population statistics. The non-parametric methods can be divided into five subclasses on the grounds of their algorithmic nature as follows:

- Similarity methods are designed to reveal gene conversion by tracking anomalous identity in variable parts of the genome [37];
- Distance methods find local dissimilarities between sequences using a sliding window technique [38];
- Compatibility methods detect phylogenetic incongruence of individual sites from alignments and do not require the phylogeny itself [39,40];
- Substitution distribution approaches group together sequences with similar patterns of integral substitution properties through comparison with the calculated model distribution [41];
- Phylogenetic methods are based on topological differences between phylogenetic trees, and they represent the most frequently used class of methods in the current studies [42,43,44].

There are three groups of methods for revealing HGT, with two of them being similar to what is applied in HR detection. [2]. The first group is represented by parametric methods, that are aimed to find genetic loci with properties that differ from the genomic average, including GC content [45], oligonucleotide spectrum [46], DNA structure modeling [47], and genomic context [48]. The second group, namely, phylogenetic methods, falls into two subcategories: explicit and implicit phylogenetic methods [2] with the former comparing trees’ topologies and the latter analyzing distances between genomes [2]. The third group examines changes in synteny, i.e., the co-localization of genetic loci in the same regions [49].

**Figure 1:**
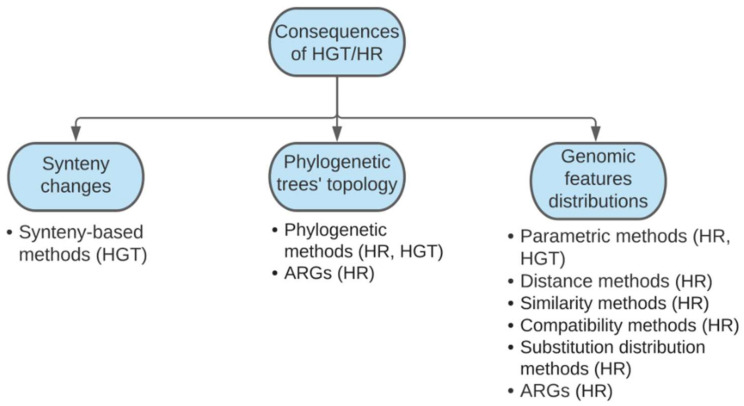
A combined classification of methods for detecting homologous recombination and horizontal gene transfer depending on the genomic consequences of the events. HR—homologous recombination, HGT—horizontal gene transfer, ARGs—ancestral recombination graphs.

As mentioned above, the interconnection between HGT and HR should not be ignored because simultaneous detection of these events can help to disentangle genome evolution. Moreover, the underlying algorithms in described methods are quite similar, and, furthermore, they actually deal with similar, but not opposite, goals, namely, finding loci subjected to recombination/transfer and calculating the frequency of such events. Different classifications do not contradict each other, thus, allowing us to unify them into a combined classification scheme based on the consequences of both HR and HGT (Figure 1). There are three possible scenarios leading to detectable signals in biological data. First, HR and HGT affect the relative positions of genes in the genome through loci gain/loss, repositioning, and duplication, thus, disrupting synteny which is especially conspicuous when comparing whole-genome sequences from diverse strains [49,50]. Second, phylogeny reconstruction based on different loci susceptible to HR or stemming from HGT would cause inconsistencies when collating different gene-based trees or comparing them to those representing species evolution [1,2]. Third, HR and HGT evoke traceable patterns of distributions of genomic properties, namely, single nucleotide polymorphisms (SNPs), alterations in GC-content, etc. [1,2,49]. While there are informative reviews discussing software coupled with guidelines to choose a particular method [1,2,3,26], recently, a lot of new tools have been devised which have not yet been systematically reviewed (Figure 1). Therefore, due to the progress in computational approaches and the occurrence of the novel tools, we discuss them in accordance with the proposed classification in the following section.

## 3. Current Bioinformatics Tools for Recombination Analysis

### 3.1. Synteny-Based Methods

Looking from the angle of genomic context, it is possible to find HGT signals in a synteny-aware way. Synteny has been defined as the degree of genomic conservation regarding the relative positions between genes [49]. Hence, changes in synteny can be traced to detect horizontally acquired genes by comparing the order of the loci in the defined genomic interval [49]. The so-called synteny index (SI) was proposed for such purposes and implemented in the Phylo SI software [51]. The synteny index denotes the number of shared gene pairs between most k genes both downstream and upstream of a selected shared ortholog. Then, the average values for all the genes within a pairwise comparison can be utilized to construct a synteny-aware phylogeny [51]. Later on, the SI was incorporated into the nearHGT tool together with constant relative mutability (CRM), another method of calculation that assumes mutation rates to remain constant for each gene within a genome [49]. For two orthologs in two species that exhibit increased similarity with other orthologs diverging in accordance with the mutability model, this approach reports a putative HGT event. Thus, in the beginning, possible HGT candidates are selected through SI calculation, and subsequently, patterns of gene divergence using CRM are defined. In the end, the chi-square test is performed to calculate the significance of the predicted events [49]. A further improvement considers the length of the transfer genes and also utilizes the Chernoff bound test instead of the chi-square test, thus, reducing the number of false-positive calls [50]. The nearHGT program has been applied to evaluate the HGT rate in *Mycobacterium leprae*, which displayed that pseudogenized loci were transferred with increased frequency in contrast to functional genes [9]. Unfortunately, the available nearHGT program only calculated the probability of HGT for a given set of sequences [49]. The prior steps of calculating the SI index and reporting possible HGT has not been provided as available scripts, thus, nearHGT is more of a conceptual method than a ready-to-use application.

Although other synteny-aware utilities do not report HGT events directly, they can indirectly point out candidates to explore. Lots of genomic browsers have been developed to visualize synteny, namely, BAGET for retrieving syntenic information for a certain gene [52], Synima to juxtapose loci between genomes [53], and SYN-View to investigate antibiotic resistance gene clusters [54]. Sibelia can obtain syntenic blocks in analyzed genomes [55], while SyntTax and SynTracker link them with taxonomical and strain-specific relationships [56,57]. Finally, current pan-genome analysis software now operates with synteny: PEPPAN enables one to retrieve putative HGT events from the accessory genes matrix through synteny-aware pangenome reconstruction [58] and Panaroo provides a graph with syntenic consecutive triplets of gene families, thus, detecting structural variations [59]. Finally, syntenic information could be obtained from gene-to-gene alignments with conventional tools [60,61].

### 3.2. Phylogenetic Methods

#### 3.2.1. Phylogenetic Methods for HR Detection

One approach to finding present recombination events is called phylogenetic networks. In as much as recombination events lead to intermingling between evolutionally distant lineages, a conventional representation of the evolution as a tree does not reflect the actual phylogenesis. Given that phylogenetic networks pose a more suitable visualization for genetic exchange, there are two distinct types of phylogenetic networks, namely explicit and implicit [62]. The advantage of the former is their interpretability as phylogenetic trees because these networks possess information about parents and recombinants. Unfortunately, explicit networks are hardly obtainable in practical terms, in so far as many recombination events do not provide signals strong enough to distinguish them from mutations, in particular, when they affect conservative genes [26]. In contrast, implicit networks display the most conflicting clades where tree topology is disturbed, demonstrating alternative evolutionary scenarios to be verified with other techniques [62].

Once potential signals are found, it becomes possible to identify breakpoints and to find chimeric sequences. The combination of phylogenetic and distance approaches has revealed these regions that possibly transferred during recombination and the disentangling evolutionary relationships between analyzed sequences regarding these genetic exchanges [26]. Dividing sequences into parts can be carried out by a static procedure with constant borders [63] or dynamically by splitting into two chunks [38], applying a sliding window [41], or more complex heuristics [64]. Parental and recombinant sequences are usually determined by analyzing phylogenetic trees built on different parts of the sequences detected during the previous step. When a potential recombination event is identified, its statistical significance is evaluated, for example, by parametric bootstrap [65] or chi-square distribution [66].

At the moment, the most frequently applied novel programs to examine homologous recombination, as well as HGT, are based on phylogenetic methods. Among these, RDP4 [66] represents a user-friendly application implementing several algorithms with different partitioning schemes for identifying recombined sequences. Its advantages include utilizing a combination of phylogenetic and distance methods providing identification of parent–child relationships and breakpoints in recombined entries [26]. Its updated version, RDP5 [67] has incorporated extra statistical tests, namely, the Φ_w_ test [39], the four-gamete test [68], and adapted versions of the homoplasy test [43]. In RDP5, run time speed has been increased up to five times and the number of analyzed bacterial genomes up to 120 times [67]. Still, it cannot handle large batches of bacterial genomes, and therefore, it has been used to trace recombination predominantly in viral genomes, for example, in porcine reproductive and respiratory syndrome virus (PRRSV) [69], SARS-CoV-2 [70], human rhinovirus [71], and feline parvovirus [72]. However, it should be noted that the algorithm inherits limitations of phylogenetic algorithms, the most evident of which is its inability to reveal distant events [26]. Thus, this tool is more suitable for identifying recent events in sequences with moderate divergence and relatively small genomic datasets.

Another group of phylogenetic tools can apply the so-called clonal model [10,44,64,73]. This approach is aimed at scanning whole-genome sequences, in which conservative loci within housekeeping genes are used for phylogeny reconstruction. The chosen genes are considered to depict a clonal frame showing direct relationships between distinct clonal groups.

Gubbins starts with removing SNPs (single nucleotide polymorphisms) that do not fit the assumption of a constant per-site mutation rate, and then places these inconsistencies among the tree built on the remaining polymorphisms [44]. Among its applications, Gubbins has harnessed visualizing and characterizing recombination in Global Pneumococcal Sequence Clusters (GPSCs) [74] and pneumococcal capsular loci [75].

ClonalFrameML uses a pre-reconstructed starting tree and calculates the probability of engaging in recombination for each site using Bayesian maximum likelihood (ML) calculations [73]. ClonalFrameML has been widely used in bacterial genetics to evaluate within-population recombination rate in *Prochlorococcus* lineages [76], *Staphylococcus aureus* strains [77], and biosynthetic gene clusters in the *Salinispora* sp. [78].

Although BratNextGen and fastGEAR are not truly phylogenetic methods, they still operate with clonal relationships, hence, it is more appropriate to discuss them in the current section. However, they do not analyze single nucleotide polymorphisms (SNPs) directly but compare the distributions of variants within clonal lineages using hidden Markov model (HMM) approaches [10,64]. Notably, the latter represents an improvement of the former with higher statistical power. The ability of BratNextGen to reveal ancestral recombination has been applied in studies related to *Streptomyces* species [79], antibiotic-resistant *Staphylococcus aureus* strains [80], and differentiated *Xylella fastidiosa* isolates [81].

On the one hand, all the programs described provide a characterization of SNPs, revealing whether they originate from mutation or recombination, which allows calculating the r/m rate (the probability that a given site stems from recombination rather than mutation) as a proportion of recombination-derived variants. Moreover, these algorithms can handle large datasets due to their high computational capacities. On the other hand, all described tools cannot efficiently distinguish recombination from mutations in the presence of disruptive selection; they also lack statistical power when analyzing highly similar sequences [36]. Another limitation lies in the reliance on phylogenetic trees obtained by methods implying no recombination. Actually, such phylogenetic trees do not portray clonal relationships between ancestors and descendants, as the topology depicts different recombinational rates in diverging bacterial populations rather than sequential evolutionary development [82]. Keeping in mind the questionable feasibility of reflecting clonality even within conservative loci [82], the validity of matching recombination events to the overall phylogeny appears to be dubious. Therefore, it seems more valid to provide per-lineage recombination frequency instead of the overall rate. To sum up, the described tools allow examining large genomic datasets. Ancestral state reconstruction allows them to reveal possible ancestral events particularly optimized in the fastGEAR algorithm [10]. Moreover, due to single-lineage-based clonal relationships, ClonalFrameML [73], Gubbins [44], and BratNextGen [64] are tuned to analyze single bacterial linage with moderate diversity, while fastGEAR harnesses studying interspecies events in sequences with higher diversity [10].

#### 3.2.2. Implicit Phylogenetic Methods to Reveal HGT

In revealing HGT events, explicit phylogenetic methods are presented by straightforward testing of topological similarity [83], decomposing trees’ initial partitions [84], pruning and regrafting subtrees [85], or selecting appropriate reconciliation models accounting for gene loss/duplication and homologous recombination events [86]. Implicit phylogenetic methods do not rely directly on juxtaposing species- and gene-based trees but summarize distances between genomes analyzed to reveal excessively related or different sequences by utilizing BLAST searches [87], disparities between species and gene distances [88], building so-called phylogenetic profiles characterizing patterns of gene presence/absence [89], and clustering polymorphisms [90]. Similar to homologous recombination, novel phylogenetic software to detect horizontal events has been devised recently. It should be noted, however, that the most current tools fall into an implicit category, therefore, these approaches are described here.

HGT-Finder implies a BLAST-based algorithm to provide a set of likely transferred sequences with a transfer index value and significance estimations [91]. The results of the BLAST search against the NCBI non-redundant protein (NCBI-nr) database are utilized to infer relative bit scores (R) calculated as a ratio of the observed bit score to the bit score of the same-sequence alignment. Simultaneously, taxonomic distance using the NCBI Taxonomy database (D) is evaluated as the number of taxonomic units in the query divided by the number of common units with the respective database hit [91]. Then, the transfer index is determined by the mean RD value for each hit genome divided by the number of genomes. Applying HGT-Finder has provided HGT screening in *Burkholderia glumae* [92] and *Aspergillus* sp. genomes [91].

HGTector is another tool depending on BLAST searches coupled with taxonomic inference. First, it categorizes genomic hits into three groups: self (the closest strains), close (the same genera or close family), and distal (other families, orders) [93]. The distributions of bit scores for the three categories are then followed by a gene-wise estimation of deviation from these distributions, indicating possible HGT-derived genes [93]. HGTector has been used to infer exchanges in *Legionella* sp. [94], *Nocardia* sp. [95], and *Blautia* sp. [92].

RecentHGT was developed to reveal HGT events between close species [96]. It performs global Needleman–Wunsch alignment of protein-coding sequences and builds the distribution accordingly. Next, particular hits are tested in terms of the inconsistency with the distribution [96]. The approach has successfully harnessed HGTs in *Rhizobium* strains [96,97].

HGT-Finder and HGTector are more sophisticated taxonomy-wise methods as compared with simple BLAST searches; however, it should be considered that they lack sensitivity as the success of detection depends on taxonomical distance [91,93]. Their design makes them more suitable for revealing HGT between distant bacterial lineages, for example, different taxonomic groups. Contrarily, RecentHGT, in its turn, is designed to detect genetic exchange in close lineages, and therefore can distinguish HGT events from highly conserved housekeeping genes with a reduced false-positive rate as compared with other tools [96].

Of the most current tools to mention, ShadowCaster represents a hybrid approach incorporating both composition-based support vector machines (SVMs) and implicit phylogenetic methods based on the phylogenetic shadow that is constructed on proteomes of species both closely related and distant to the analyzed ones [98]. ShadowCaster shows improved sensitivity as compared with other methods, and moreover, it can detect both close and distant events. For instance, it revealed the transfer of heavy metal resistance genes in *Rhodanobacter denitrificans* with high accuracy [98]. Nevertheless, while it looks promising, it does not reflect the direction of transfers [98]. As it was not benchmarked by comparing with RecentHGT, it is impossible to state which tool shows better performance, nevertheless, it could be proposed that due to a hybrid check implemented, ShadowCaster may be more sensitive and accurate.

### 3.3. Methods Based on Genetic Features

#### 3.3.1. Compatibility Methods to Reveal HR

Being non-phylogenetic, compatibility methods now seem of great potential due to their ease and computational effectiveness. The basic approach of such evaluations is a so-called ”four-gamete test” [68]. If two sites provide a genealogy that should involve recurrent mutations to resolve evolutionary relationships, then, these sites are called phylogenetically incompatible, implying their occurrence through homoplasy or recombination [68]. In practice, it is almost impossible to tell recombination from homoplasy for highly similar sequences; nonetheless, one can summarize all homoplasic features and can compare results with the predictions of the model recombination-free distribution [3]. The most commonly used implementations of this approach are the homoplasy test [43] and its improvement, a Φ_w_ test [39], both depending on the frequency and distribution of incompatible sites.

The recently developed ptACR program identifies potential breakpoints with a sliding window followed by a permutational test to calculate the significance of found events [40]. Its architecture has ensured robustness to false-positive results checked on clinical isolates of *Staphylococcus aureus* [40] Nonetheless, ptACR’s disadvantage is the absence of strategies to handle gaps; thus, it is hard, if possible, to analyze divergent sequences with this utility [40], that is to say, this program is useful if the aim of the research is to reveal the most probable recombination events in sequences with moderate diversity.

#### 3.3.2. Substitution Distribution-Based HR Detection Approaches

Similar to compatibility approaches, substitution distribution methods have regained attention due to their high speed as compared with phylogenetic approaches. HREfinder is a dynamic algorithm that divides the genome into blocks where each polymorphism is estimated to result from mutation, homologous recombination, or sequencing error [99]. The stepped validation guarantees obtaining events with high probability as tested in a *Xanthomonas oryzae* evolution study [100]. The sensitivity of HREfinder continuously grows with sequence diversity, while at the same time, a false-positive rate is coupled with it [99]. Hence, HREfinder just like ptACR, is suitable when dealing with moderately divergent sequences. Within the optimal diversion range, HREfinder detects mostly true events, however, it also tends to miss a lot of them because of detection thresholds [99].

#### 3.3.3. Parametric Methods for HR Identification

Parametric methods are mostly aimed at evaluating the overall HR rate based on population genetics principles. [3]. Population recombination rate (*p*) is calculated as $p=4N_{e}*r$, where *N_e_* is the effective population size and r stands for per-site recombination rate for one generation. Similarly, the population mutation rate is determined by the following equation: $\theta =4N_{e}*µ$, where µ denotes per-site mutation rate. The *p/θ* ratio is considered to be an average quantitative variable characterizing recombination for a particular population [1].

One program implementing these methods is Mcorr [101]. This tool calculates the correlation of synonymous substitutions (correlation profiles), and the average recombination rate is delineated on the basis of these profiles [101]. The authors denoted a correlation profile as the probability of observing a difference at the *i + l* site for a randomly chosen site *i*, where *l* is the distance in nucleotides. The function *P*(*l*) is constant in the absence of recombination, whereas the presence of recombination causes a monotonic decrease of the *P*(*l*) function [101]. The method is highly useful in metagenomic studies, for example, subpopulations in soil metagenome [102] or multidrug-resistant *Escherichia coli* ST131 populations in the infant gut microbiome [101]. The presented statistic provides a vivid interpretable result reflecting the recombination rate, however, the congruity between this method and compatibility-based HR frequency calculation has not been assessed yet.

#### 3.3.4. Ancestral Recombination Graphs

A distinct method combining phylogenetic incongruence detection, population genetics principles of coalescent theory, and phylogenetic networks is a reconstruction of so-called ancestral recombination graphs (ARGs) [63]. The ARG represents a directed graph in which the most probable site-to-site relationships are exhibited, thus, enabling lateral connections denoting horizontal events such as recombination, which is distinct from classic trees with acyclic topology determined by the average identity between sequences [63]. Being a hybrid approach, ARG construction can depict evolutionary histories that involve recombination coupled with the timed presentation of vertical inheritance, thus, providing a detailed evolution-wise report of recombination events [26].

Bacter, a Bayesian algorithm, has been applied to reconstruct ARGs based on the ClonalOrigin model and Markov chain Monte Carlo (MCMC) algorithm that are used jointly to infer genealogical relationships as well as homologous conversion events and the overall conversion rate [35]. This single-step procedure, instead of a stepped algorithm, improves detection and reduces uncertainty in the case of a poor phylogenetic signal [35]. Its application has accurately revealed previously undetected gene flow between pathogenic and nonpathogenic *Escherichia coli* serotype O157 representatives [35]. Still, the limitation of this tool is its dependence on a lot of parameters to be optimized for each study, poor throughput, and inefficiency if analyzing long genomes, especially big batches [34].

To handle the inference of ARGs on a large genomic scale, a computationally efficient alternative has been proposed. This approach is called topological data analysis (TDA) in which genomes are treated as points in a high dimensional space with pairwise distances delineated by genetic dissimilarities [103]. Loops in this space linking points occur in the presence of recombination, hence, summarizing loops generate a structure closely related to ARGs, namely, topological ARG (tARG) that depicts minimal recombination histories [103]. TARGet was designed in accordance with the aforementioned principles. While it was tested on eukaryotic organisms, it seems to be applicable for analyzing bacterial genomes, especially when examining large datasets [103]. Topological data analysis is a promising approach regarding its computational effectiveness, although tARG itself cannot depict the specific evolutionary histories behind the data [103]. Therefore, an available tool for recombination-wise bacterial evolution reconstruction, Bacter, is reasonable to apply when dealing with small genomes or parts of genomes, thus, it is necessary to develop computationally efficient tools possibly based on the principles of topological data analysis.

#### 3.3.5. Parametric Methods for Finding HGT Events

Sample-based parametric methods in the context of an HGT analysis have been considered to be less accurate than phylogenetic methods which are dominant in the repertoire of HGT detection programs; however, recently, novel tools with better performance have been devised. They have been applied to obtain the most probable HGT-subjected parts of the genome and the overall transfer frequency. The respective HGT-rate computations rely on the calculation of the HGT-affected genome fraction [104], the ratio of gene gain to gene loss [105], or the total number of detected HGT events divided by the total number of compared genomes [106,107].

To reveal HGT-subjected parts, sequence clustering methods seem to be a perspective approach to deal with the constraints of current tools. The Clusterflock algorithm utilizes a model of self-organizing swarm intelligence originally proposed to imitate bird and insect behavior [108]. This model enables clustering based on a distance matrix with arbitrary distance metrics. The comparison of orthologous gene families’ (OGFs) clusters with obtained flocked clusters has revealed signals of HGT between sequences. Its application has disentangled a large-scale map of genetic exchanges in *Staphylococcus aureus* [108], still, the Clusterflock has not been benchmarked in the context of comparison with other tools or calculating accuracy and specificity.

The genome mosaic structure (gmos) algorithm was developed to overcome difficulties related to computational costs of full genome-comparison alignments [109]. This program performs local alignments for a given query sequence against subject genomes, refines the alignments according to the substitution models, and finally, overlaps the refined local alignments to gain the mosaic structure of the regions. The utility has been used to track mosaic sequences in the pathogenic *Enterococcus faecium* strain [109]. The advantage of such an approach is the ability to reveal both homologous recombination events and horizontally transferred genes. However, the latter is possible only if genomes possess sufficient similarity in transfer regions; moreover, the tool does not resolve the direction of transfer/exchange [109].

GeneMates is an R package to reveal co-transferred genes in bacterial genomes associated with mobile genetic elements [110]. In the package, the matrix of core genome SNPs coupled with allelic presence/absence matrix is analyzed using linear mixed models to generate a network of alleles that are most likely co-transferred together. This framework transcends simple co-occurrence tests according to a validation study of GeneMates on known antibiotic resistance genes in *Escherichia coli* and *Salmonella* Typhimurium; nonetheless, it is designed for a specific aim to identify intraspecies events, while its dependence on core SNPs may probably restrict the sensitivity of the analysis [110].

The abovementioned tools rely on completed and maximally annotated genomes. In contrast to it, Daisy is a reference-free method that processes short reads to detect HGT boundaries via split-read mapping and coverage information, and it leads to outperforming assembly-based approaches [111]. Its performance has been checked on a simulated *H. pylori* dataset and two real *E. coli* datasets [111]. While providing high sensitivity, Daisy relies on short reads only and requires genomes with explicitly defined suspected donor and acceptor, thus, it is not applicable to process long reads or it cannot compare bathes of genomes when donor and acceptor are unknown.

## 4. Assessing the Effectiveness of Recombination Detection Software

To choose a particular algorithm to detect HR and HGT in biological data, it is useful to understand the expected rate of false-positive calls. Erroneous identification of recombination events may occur when analyzing extremely divergent sequences, given that in the tools applied, statistical power proportionally increases with sequence divergence [112]. However, handling substantially similar strains may also generate errors [113]. Some methods are also sensitive to asymmetric tree topology [112]. If linkage disequilibrium between nucleotide substitutions is used to predict recombination events, findings may actually represent evolutionary selection signals instead of genetic exchange [114]. A so-called “patchy-tachy” (PT) phenomenon describes sequences in which different partitions exhibit unequal evolution rates, which leads to an excess in false-positive results [115]. Tracking HGT can generate false-positive results as well. For instance, parametric methods based on codon usage are prone to a high rate of both false-positive and false-negative results [116]. In addition, similar to HR, false-positive HGT signals likely occur if comparing closely related strains [49]. Another essential source of misreported events relates to genomic data collection, namely, assembly procedures and PCR-gained chimeric sequences. For example, a comparative study of *Mycobacterium tuberculosis* genomes revealed that most of the recombination events described in the literature were artifacts [117]. They occurred due to inconsistencies in the genomic alignments in the case of reference-based genome assembly relying on the reference assembly already containing false-positive results; hence, in bacterial genomics, high-quality de novo assemblies should be preferred instead [117]. Sample preparation could provoke artificial recombination events both during PCR amplification and data analysis of sequencing data leading to the emergence of chimeric sequences [118,119]. These chimeric sequences are often presented in current databases, thus, making it difficult, if possible, to estimate the number of artefactual data possibly utilized as reference sequences in phylogenetic studies [26].

Given a great variety of cases in which correct detection of HGT and HR is hampered (Table 1), the limits of applications for the programs have to be quantitatively evaluated to ensure choosing the most accurate and sensitive algorithms. Therefore, it seems surprising that there is a lack of comparative analyses. In most cases, such studies include only a small number of algorithms to display the performance of the recently devised tool [10,44,98], whereas comprehensive examinations currently seem outdated [112,120]. Still, for such performance tests, one can apply genome evolution simulators under HR, such as SimBac [121] and Bacmeta [122]. Nevertheless, it should be borne in mind that these simulators are coalescent-based, implying a constant recombination rate and modeling neutral evolution. In contrast, cutting-edge technologies such as CoreSimul [123] include stochastic parameters imitating environmental changes accompanied by recombination. Similar to it, there are HGT simulators such as HgtSIM [124]. Finally, the most promising simulators capable of modeling both recombination and horizontal exchange such as SLiM [125] can be utilized to jointly analyze the detection of both HR and NHR, thus, providing a comprehensive evaluation of the genetic exchange map between bacterial populations.

## 5. Conclusions

Homologous recombination (HR) and horizontal gene transfer (HGT) in bacteria are fundamental mechanisms of their evolution, and these two processes are inextricably connected on a genomic scale. HR provides allelic diversity and causes genetic gain/loss [13]. It may well maintain genome stability by discarding unused HGT-obtained genes, and sometimes this gene loss intensity does not correlate to the overall HR rate [127]. HR and HGT are of importance for fundamental science and practical application. Therefore, genomic studies require special tools for the effective detection of these events. Recently, a host of programs have been devised, and the development is still going on. Having reviewed novel bioinformatics tools, we revealed that methods depend on the consequences of HR and HGT such as alterations in synteny, trees’ topologies incongruence, and altered distribution of genetic features (Figure 1). A great variety of available programs presents dozens of applications for studies with different goals and varying performances when used on diverse data. Programs such as Mcorr [101] or clonal frame model-based tools [44,64,73] can calculate overall HR rate, while nearHGT can evaluate HGT rate [49]. ARGs implemented in Bacter [35] are tuned to depict site-wise individual HR histories, thus, being computationally expensive, sensitive to divergence, and applicable for analyzing small sets of related genomes. Parent–child relationships for large blocks are also provided by RDP4/5 [66,67] in the case of HR, and similar donor-acceptor HGT directions could be identified with Daisy [111]. The tools also differentiate in preferred data to process. ClonalFrameML [73], Gubbins [44], and RDP4/5 [66,67] manage to detect recent HR events in moderately divergent sequences, while fastGEAR [10] is suitable for digging ancestral and recent recombination events in sequences with high divergence. If highly accurate detection of true recombination events is needed, ptACR [40] and HREfinder [99] seem to be useful, while, at the same time, they lack sensitivity. Similar to HR, RecentHGT [96] shows a lower false-positive rate being appropriately utilized to uncover recent transfers in similar sequences, whereas HGT-Finder [91] and HGTector [93] are tuned to trace events in distant genomes. Similar to fastGEAR [10], ShadowCaster [98] predicts both distant and close HGT events and potentially appears to be the most effective HGT-detecting tool by far. To sum up, state-of-the-art approaches for studying HR and HGT are characterized by different sensitivities and accuracies, and they find either recent or ancient events in similar, moderately different, or highly divergent sequences. We might conclude, that the tools reviewed show better performance when detecting some types of recombination events while being less effective to reveal others. Therefore, it looks promising to develop new software that incorporates hybrid approaches to improve recombination detection. Going further, given the genomic interrelation between HR and HGT affecting each other in terms of frequency and direction, a comprehensive framework equipped with both HR and HGT predictors would sufficiently broaden our understanding of the mechanisms driving the plasticity of bacterial genomes.

## Figures and Tables

**Table 1:** Current bioinformatics tools for detecting homologous recombination and horizontal gene transfer in genetic data. The table summarizes tools’ properties in terms of algorithms applied, input files and output results, type of detected events, advantages, and limitations.

Tool	Applied Approach	Method’s Class	Input	Output	Detected Events	Advantages	Limitations	References
*Homologous Recombination (HR) Identification*								
RDP4/RDP5	Combination of phylogenetic and distance methods	Phylogenetic and distance-based	Alignments in FASTA format	Recombination events with phylogenetic relationships and breakpoints coordinates for chimeric sequences in tabular format	Recent	Robustness and providing the information on the direction of exchanges	Inability to reveal distant events and high computational costs	[66,67]
Gubbins	Revealing increased substitution rate among ML-tree branches	Phylogenetic	Alignments in FASTA format	Coordinates of recombination events tabular format and their visualization on the genome alignment	Recent and ancestral	Precise reconstruction of ancestral state	High computational costs and possible false-positive results when analyzing trees with short branches (theoretically)	[44]
ClonalFrameML	Maximal likelihood-based clonal model	Phylogenetic	Alignments in FASTA format and guiding tree	Phylogeny regarding recombination and visualization of events’ coordinates on the genome alignment in tabular format	Recent and ancestral	Computational effectiveness	Underestimation of recombination rate in datasets with intensive recombination	[73]
BratNextGen	Bayesian modeling	Substitution distribution	Alignments in FASTA format	Coordinates of the events in tabular format and visualization of transmitted regions on the genome alignment	Recent and ancestral	Computational effectiveness	False-negative results in the case of mosaic sequences with multiple recombination events	[126]
fastGEAR	HMM algorithms coupled with Bayesian clustering	Substitution distribution	Alignments in FASTA format	Coordinates of ancestral and recent recombination events in tabular format	Recent and ancestral	Computational effectiveness, high sensitivity, and handling of missing data	Missing events between closely related species	[10]
ptACR	Genome-wise average SNP compatibility calculation	Compatibility	Gap-free alignments in PHYLIP format	Genomic coordinates of recombination events in tabular format	Recent	High accuracy and robustness to false-positive results	Inability to process alignments with gaps and high false-negative rate when processing divergent sequences	[40]
HREfinder	Genome partitioning into SNP-flanked blocks	Substitution distribution	Genomes in FASTA format, tree in Newick format, and SNP list in tabular format	List of sequences subjected to recombination in tabular format	Recent	High accuracy	High false-negative rate when processing divergent sequences	[99]
mcorr	Building correlation profile of synonymous substitution	Parametric	Alignments in XMFA or BAM formats	Tables and figures depicting the average recombination rate	The total rate of recent/ancient events	The ability to process raw reads and metagenomic data	Has not been compared to conventional r/m rate calculating tools	[101]
Bacter	Markov chain Monte Carlo (MCMC)	ARG	Alignments in FASTA format	Ancestral recombination graph (ARG) in Newick format	Recent	Improved detection of the events in the case of poor phylogenetic signal	Dependence on predetermined parameters and high computational costs	[35]
TARGet	Topological data analysis (TDA)	ARG	Alignments in FASTA format without gaps or segregating sites denoted by 1 and 0	Ancestral recombination graph (ARG) in XML format and positions of reticulate events	Recent	Computational effectiveness	Inability to process alignments with gaps	[103]
*Horizontal Gene Transfer (HGT) Detection*								
Clusterflock	Self-organizing flock algorithm	Parametric	Sequences and a distance matrix	Clusters of sequences in tabular format	Recent	Applicability to any distance metrics and resilience to missing data	Has not been compared to the existing tools	[108]
gmos	Pairwise local alignments with subsequent regions overlapping	Parametric	Query and subject genomes in FASTA format	Structural variants in FASTA format	Recent	Computational effectiveness and the ability to reveal both HR and HGT	Depends heavily on the high similarity between transferred regions	[109]
GeneMates	Association tests with the linear-mixed model accounting for population structure	Parametric	Genome assemblies in FASTA format and raw reads in FASTQ format	The linkage network of horizontally co-transferred alleles in tabular format	Recent	Resolving co-occurred HGT events	Reduced sensitivity due to the dependence on core SNPs	[110]
ShadowCaster	Support vector machine-based hybrid approach	Implicit phylogenetic and parametric	A query genome and proteome and list of related proteomes in FASTA format	The list of HGT candidates with corresponding likelihood calculations in tabular format	Recent and ancestral	High sensitivity when reveling both recent and ancient events and reduced false-positive rate	Does not determine the directions of transfers and processes only a single genome	[98]
nearHGT	Calculating synteny index (SI) followed by constant relative mutability (CRM) measurement	Synteny-based and parametric	Reference and putatively transferred sequences in FASTA format	Chi-square-based *p*-value denoting the probability of HGT	Recent	High sensitivity	No ready-made application is available	[49]
HGT-Finder	Similarity ratio evaluation for proteins according to BLAST hits and taxonomic distance calculation based on the NCBI Taxonomy annotation	Implicit phylogenetic	The BLAST search result and the NCBI Taxonomy database	Tabular format file with the transfer index value for a protein	Recent	Detecting mostly true events	High reliance on the taxonomic nomenclature and low sensitivity	[91]
HGTector	Analyzing BLAST hit distribution patterns according to predefined evolutionary categories	Implicit phylogenetic	FASTA files of amino acid sequences for each analyzed genome	List of candidate HGT-derived genes with the respective silhouette scores in tabular format	Recent	Insensitive to gene loss, rate variations, and database errors	High reliance on the taxonomic nomenclature and low sensitivity	[93]
RecentHGT	The expectation-maximization algorithm based on the sequence-similarity distribution of orthologous genes	Implicit phylogenetic	Tabular file with strains information and RAST-annotated GenBank file	Putative HGT events in chromosomal and plasmid regions in tabular format	Recent	Reduced false-positive rate when processing conserved genes	Missing events when analyzing divergent sequences	[96]
Daisy	Mapping-based detection relying on short read pairs and coverage information	Parametric	Reads from the analyzed organism and poposed acceptor and donor genomes in FASTA format	A variant call format (VCF) file reporting HGT candidates meeting the predefined threshold and tabular format file with all potential events	Recent	Outperforms reference genome-based approaches if short reads are available	Requires short reads only and explicit specifying recipient and donor genomes	[11]

## Data Availability

Not applicable.

## Abbreviations

HR	Homologous recombination
NHR	Non-homologous recombination
HGT	Horizontal gene transfer
LGT	Lateral gene transfer
SSAPs	Single-strand annealing proteins
SSA	Single-strand annealing
ARGs	Ancestral recombination graphs
MLST	Multilocus sequence typing
CGP	Coarse-graining approach for phylogenetic reconstruction
CRM	Constant relative mutability
SI	Synteny index
PRRSV	Porcine reproductive and respiratory syndrome virus
GPSCs	Global pneumococcal sequence clusters
SNPs	Single nucleotide polymorphisms
HMM	Hidden Markov model
TDA	Topological data analysis
MCMC	Markov chain Monte Carlo
tARG	Topological ARG
OGFs	Orthologous gene families
